# MAFB is essential for the maintenance of adult human α-cell identity and glucagon secretion

**DOI:** 10.64898/2026.08.08.743687

**Published:** 2026-08-14

**Authors:** KC Coate, J Liu, M Guo, X Tong, VMN Coykendall, C Harmelink, N Dey, G Reynolds, N Mohanty, RE Jenkins, R Aramandla, JP Cartailler, AC Powers, PE MacDonald, SK Kim, RW Stein

## Abstract

Dysregulated hormone secretion and erosion of endocrine cell identity are features of type 1 and type 2 diabetes, but the transcriptional programs maintaining adult human islet identity and function remain poorly defined. The large MAF transcription factor MAFB is expressed in human α- and β-cells, marks their most functionally mature subpopulations, and is downregulated in diabetes, but its role in adult human islets has not been tested directly. Using shRNA-mediated MAFB knockdown (KD) in whole and CD26 ^+^ α-cell-enriched human pseudoislets, we found that whole pseudoislet MAFB KD impaired glucagon synthesis and secretion while only modestly reducing insulin content and cAMP-potentiated insulin release. Single-cell profiling detected no β-cell transcriptional response beyond MAFB KD itself, consistent with buffering by the related β-cell-enriched MAFA transcription factor. In contrast, α-cell-restricted MAFB KD unmasked a cell-autonomous requirement for MAFB in stimulus-secretion coupling. MAFB deficiency also destabilized α-cell identity, downregulating canonical α-cell and neuroendocrine secretory genes while ectopically inducing mesenchymal and extracellular matrix remodeling programs. In addition, MAFB-dependent downregulation of electron transport chain genes was confined to a large α-cell subcluster, manifesting as impaired islet-wide mitochondrial respiration within the broader α-cell population. Together, these findings identify MAFB as an essential adult human α-cell maintenance factor that links diabetes-associated downregulation to impaired glucagon secretion, α-cell identity erosion, and mitochondrial dysfunction.

**RESEARCH IN CONTEXT:**

- **What is already known about this subject?**
  - MAFB is expressed in adult human α- and β-cells, marks their most functionally mature subpopulations, and is downregulated in type 1 and type 2 diabetes
  - In human stem cell models, MAFB is essential for generating insulin-producing β-like cells, whereas glucagon-producing α-like cells are reduced but still formed
  - Neither model addresses adult human islets: rodent MafB becomes α-cell restricted after birth, and stem cell models capture differentiation, not maintenance
- **What is the key question?**
  - Is MAFB required to maintain identity and secretory function in adult human islet cells?
- **What are the new findings?**
  - MAFB knockdown in primary human pseudoislets impaired glucagon synthesis and secretion but minimally affected β-cells, consistent with buffering by MAFA
  - Knockdown in CD26+ α-cell-enriched pseudoislets revealed a cell-autonomous requirement for MAFB in stimulus-secretion coupling, and destabilized α-cell identity by inducing mesenchymal and extracellular matrix programs
  - MAFB loss downregulated electron transport chain genes in the largest α-cell subcluster and reduced mitochondrial respiration
- **How might this impact on clinical practice in the foreseeable future?**
  - Preserving MAFB activity in adult human α-cells may represent a strategy to limit α-cell dysfunction in diabetes

## Full Text Availability

The license terms selected by the author(s) for this preprint version do not permit archiving in PMC. The full text is available from the preprint server.

